# Aortic Stiffness Measured from Either 2D/4D Flow and Cine MRI or Applanation Tonometry in Coronary Artery Disease: A Case–Control Study

**DOI:** 10.3390/jcm12113643

**Published:** 2023-05-24

**Authors:** Lan-Anh Nguyen, Sophia Houriez-Gombaud-Saintonge, Etienne Puymirat, Umit Gencer, Thomas Dietenbeck, Kevin Bouaou, Alain De Cesare, Emilie Bollache, Elie Mousseaux, Nadjia Kachenoura, Gilles Soulat

**Affiliations:** 1 Université Paris Cité, PARCC, INSERM, F-75015 Paris, France; 2 Laboratoire d’Imagerie Biomédicale, Sorbonne Université, INSERM, CNRS, F-75006 Paris, France; 3 Assistance Publique Hôpitaux de Paris, Hôpital Européen Georges Pompidou, F-75015 Paris, France

**Keywords:** MRI, PWV, 4D flow, coronary artery disease

## Abstract

Background and objective: Aortic stiffness can be evaluated by aortic distensibility or pulse wave velocity (PWV) using applanation tonometry, 2D phase contrast (PC) MRI and the emerging 4D flow MRI. However, such MRI tools may reach their technical limitations in populations with cardiovascular disease. Accordingly, this work focuses on the diagnostic value of aortic stiffness evaluated either by applanation tonometry or MRI in high-risk coronary artery disease (CAD) patients. Methods: 35 patients with a multivessel CAD and a myocardial infarction treated 1 year before were prospectively recruited and compared with 18 controls with equivalent age and sex distribution. Ascending aorta distensibility and aortic arch 2D PWV were estimated along with 4D PWV. Furthermore, applanation tonometry carotid-to-femoral PWV (cf PWV) was recorded immediately after MRI. Results: While no significant changes were found for aortic distensibility; cf PWV, 2D PWV and 4D PWV were significantly higher in CAD patients than controls (12.7 ± 2.9 vs. 9.6 ± 1.1; 11.0 ± 3.4 vs. 8.0 ± 2.05 and 17.3 ± 4.0 vs. 8.7 ± 2.5 m·s^−1^ respectively, *p* < 0.001). The receiver operating characteristic (ROC) analysis performed to assess the ability of stiffness indices to separate CAD subjects from controls revealed the highest area under the curve (AUC) for 4D PWV (0.97) with an optimal threshold of 12.9 m·s^−1^ (sensitivity of 88.6% and specificity of 94.4%). Conclusions: PWV estimated from 4D flow MRI showed the best diagnostic performances in identifying severe stable CAD patients from age and sex-matched controls, as compared to 2D flow MRI PWV, cf PWV and aortic distensibility.

## 1. Summary

In the setting of high-risk coronary artery disease, pulse wave velocity estimated by 4D flow was able to differentiate patients from controls better than other stiffness assessments.

## 2. Key Results

In this prospective study, which included 35 patients with severe CAD 1 year from a myocardial infarction and 18 matched controls, the measurement of aortic stiffness by Pulse wave velocity using 4D flow showed the best sensitivity and specificity to differentiate patients from controls compared to 2D flow, MRI aortic distensibility and carotid-to-femoral pulse wave velocity.

Four-dimensional flow-derived pulse wave velocity was related to hypertrophic remodeling (ρ = 0.28; *p* = 0.048).

## 3. Introduction

Aortic stiffness, reflecting the loss in arterial elastic function, increases with aging and, in the case of atherosclerotic diseases, including coronary artery disease. Pulse wave velocity (PWV), the pulse or flow waveform velocity along the arterial tree, is one of the major metrics to clinically assess arterial stiffness. Carotid-to-femoral PWV (cf PWV), evaluated by applanation tonometry, has been largely validated as an independent prognostic factor of cardiovascular morbidity and cardiovascular or all-cause mortality in the general population or in hypertensive patients [1,2,3,4,5] and is thus proposed as an additional marker of cardiovascular risk [6].

PWV can also be derived from MRI images, with the advantages of focusing on central aorta and overcoming the geometrical approximation of the arterial length, covered by the traveling waveform. Two-dimensional phase contrast (PC) can evaluate PWV within the aortic arch [3,7,8,9] with good agreement with invasive measurements [10], and it has been validated as a marker of vascular aging and an independent factor of cardiovascular morbidity [11] or mortality [12,13]. Aortic distensibility is another widely used MRI estimate of proximal aorta stiffness. It is a local measurement calculated as a combination of cine MRI-derived aortic strain and pulse pressure. It has also been validated as a marker of vascular aging and as an independent prognostic factor of cardiovascular mortality [14,15].

Four-dimensional PC MRI or 4D flow is an emerging technique to noninvasively assess PWV, with the advantage over 2D PC MRI of allowing a full aortic anatomical coverage with three-dimensional blood flow velocity encoding [16,17,18,19]. Thanks to this amount of data and coverage, several approaches have been proposed in recent years to measure PWV with validation against either cf PWV, 2D phase contrast, aortic distensibility, aging or left ventricle remodeling, mainly in cohorts of healthy volunteers or patients with aortic diseases [17,18,20,21]. However, few studies focused on patients with atherosclerotic disease [16,22,23]. Measurements in patients with vascular diseases entail further challenges: as PWV rises, a high temporal resolution is needed and aortas with complex geometry could be more difficult to assess. These concerns might be overcome by the availability of 3D segmentation techniques [24] and time-frequency domain methods for transit time [25], which make PWV assessment less sensitive to temporal resolution [21]. Since patients with confirmed atherosclerotic disease are expected to have higher aortic stiffness than age and sex-matched controls, we hypothesize that 4D flow PWV may be the best tool to assess this difference.

This work focuses on the evaluation of aortic stiffness from 4D flow MRI compared to other MRI techniques and applanation tonometry in a population of patients with coronary artery disease (CAD) and healthy controls.

## 4. Methods

### 4.1. Population

Stiffness data were collected prospectively from a subpopulation of the FLOWER MI multicentric study [26] (clinicaltrials.gov accessed on 23 April 2023 identifier: NCT02943954) to achieve this ancillary work. Briefly, the main study aimed at determining the value of the angiographic FFR (fractional flow reserve) in revascularization strategy in a population of patients with myocardial infarction and multicoronary lesions. Inclusion criteria of the main study were: STEMI patients ≥18 years old with successful culprit lesion percutaneous coronary intervention (PCI: primary, rescue or pharmaco-invasive) and ≥50% stenosis judged amenable to PCI in at least one additional nonculprit lesion. Exclusion criteria were: patients in cardiogenic shock, patients with multivessel disease transferred to surgery for coronary artery bypass graft or treatment of acute complications, previous coronary bypass surgery, extremely tortuous, calcified coronary vessels or chronic total occlusion, life expectancy <2 years, known hypersensitivity to adenosine, pregnancy, and participation to another therapeutic interventional study at the same time or within 3 months prior to the beginning of the present study. As part of this protocol, these patients could undergo a cardiac MRI after one year of follow-up. In our center, the cardiac MRI included an aortic stiffness assessment.

**Figure 1:**
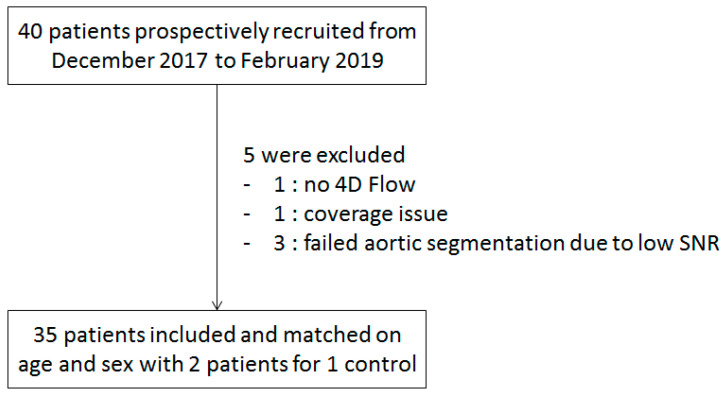
Patient selection flow diagram. SNR: signal−to−noise ratio.

The first 40 patients were recruited for this study from December 2017 to February 2019. Of these patients, 1 did not undergo the 4D flow sequences and 4 datasets were judged nonanalyzable: 1 due to a coverage issue and 3 due to low SNR leading to failure of the aortic segmentation (see below). Finally, 35 patients were analyzed and retrospectively matched on age and sex with two patients for one control (Figure 1). Controls were selected from a database of 60 healthy subjects from the Elasto-Cardio study [27] (clinicaltrials.gov accessed on 23 April 2023 identifier: NCT02537041). The study was conducted in accordance with the Declaration of Helsinki, and approved by the Ethics Committee of Comité de Protection de Personnes Ile de France (protocol FLOWER-MI, approval on 14 April 2016); written informed consent was obtained from all participants. 

### 4.2. Acquisition Protocol and CMR Data Analysis

A CMR exam was performed by using a 3 Tesla system (Mr 750W, GE Healthcare Milwaukee, WI, USA, upgraded to Signa Architect) with a 32-channel cardiac-phased array surface coil. Precontrast cine Standard Steady-State Free Precession (SSFP) sequences were acquired in axial, long-, and short-axis views during breath hold to cover the whole left ventricle [9]. Stacks of SSFP cine short-axis images were analyzed using cvi42 (Circle, Calgary, AB, Canada) to estimate LV volumes, mass and function.

#### 4.2.1. Cf PWV

Applanation tonometry of the right carotid artery and right femoral artery was performed immediately after MRI acquisitions using a Complior Device (ALAM medical, Saint Quentin Fallavier, France). Cf PWV was not feasible in 2 patients due to failure to record the femoral pressure waveform in a context of high BMI.

#### 4.2.2. Aortic Distensibility and 2D PC PWV

An SSFP cine aortic slice was acquired perpendicularly to the ascending and descending aorta at the level of the pulmonary artery bifurcation, during a breath hold (20 s of acquisition time) with a pixel size of 0.74 mm × 0.74 mm and slice thickness of 8 mm. Systolic (Amax) to diastolic (Amin) change (ΔA) in the aortic area was estimated through time-resolved automated segmentation of aortic lumen using the Artfun Software (INSERM U 1146, Paris, France) [10,28]. During the MRI exam, central aortic pressures were derived from brachial pressures with a Sphygmocor xcel device (Atcor, Naperville, IL, USA) using a previously validated generalized transfer function [28,29]. Distensibilities of the ascending and descending aortas were calculated as ΔA/(Amin × PP), where PP is the central pulse pressure and then averaged.

Two-dimensional flow imaging with through-plane velocity encoding was applied at the same anatomical location as the aortic cine acquisition during a breath hold (20 s of acquisition time, with an acquired temporal resolution of 20 ms reconstructed into 10 ms, a pixel size of 1.24 × 1.24 mm², and slice thickness of 6 mm). Such velocity data were used to calculate the ascending to descending aorta transit time using the Artfun software (INSERM U1146, Paris, France) [7,14]. A 3D centerline of the aorta was obtained from a 3D phase contrast angiogram (PC-MRA) generated around the systolic peak of the 4D flow dataset. Two-dimensional PWV was calculated as the combination of the ascending to descending aorta transit time with the corresponding aortic length measured from the 3D centerline.

#### 4.2.3. 4D Flow PWV

**Figure 2:**
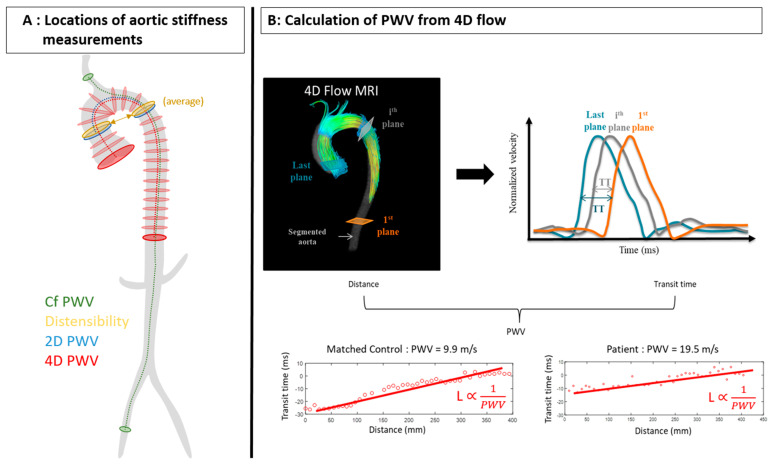
(**A**): Measurement locations for the 4 aortic stiffness estimates. (**B**): Calculation of PWV with aortic 4D flow−rate curves obtained along the centerline using the wavelet method to estimate transit time.

An ECG synchronized 4D flow CMR acquisition (10 min of acquisition time in free breathing) was performed either without or after gadolinium injection in a sagittal volume orientation encompassing the thoracic aorta. The pulse sequence consisted of a gradient echo sequence with three-directional velocity encoding (encoding velocity = 250 cm·s^−1^ for all directions). Scan parameters were as follows: average spatial resolution = 2.0 × 2.4 × 1.5 mm^3^, flip angle 7 to 15°, RT = 4.2 ms, TE = 1.60 ms, bandwidth = 62 kHz, views per segment = 2, resulting in an effective temporal resolution of 34 ms, reconstructed into 50 cardiac phases after using view sharing, independent of the value of the heart rate, acceleration factor 4, using kt-ARC. The eddy current correction, aortic segmentation and PWV computation were performed semiautomatically using a custom process, recently described [21,30]. Briefly, the 3D phase contrast angiogram (PC-MRA) generated around the systolic peak of the 4D flow dataset was used for 3D semiautomated aortic segmentation [31,32] based on 8 initial anatomical landmarks: aortic valve, sino-tubular junction, ascending aorta at the 2D slice level, brachiocephalic artery bifurcation, isthmus, descending aorta at the 2D slice level, diaphragmatic aorta and coeliac trunk. Such segmentation provided aortic length and was further used to estimate aortic flow-rate curves in consecutive planes perpendicular to the aortic centerline. Finally, aortic length was combined with transit times calculated from these latter flow-rate curves while using the wavelet-based method and considering the last abdominal plane as the reference for calculations (Figure 2) [21].

### 4.3. Statistical Analysis

Baseline characteristics were provided as mean ± SD or median [inter quartile range] as appropriate. Normality was tested using the Shapiro-Wilk test. Unpaired student t test and Wilcoxon test were used to compare patients and controls using continuous variables with Gaussian and non-Gaussian distribution, respectively, and Fisher’s exact test was used for categorical variables.

Statistical differences between the different values or aortic stiffness were analyzed using paired t tests or Wilcoxon signed rank test as appropriate. Univariate correlations between aortic stiffness measurements were reported using Pearson’s or Spearman’s correlation coefficient, as appropriate. The ability of the calculated aortic stiffness parameters to separate patients from controls, in terms of sensitivity and specificity, was evaluated using a receiver operating characteristic (ROC) analysis with definition of the optimal threshold. The correlations of aortic stiffness measurement with clinical or MRI parameters were obtained using Pearson’s or Spearman’s coefficient, as appropriate. A *p* value < 0.05 was used to indicate statistical significance. Analyses were performed using JMP 14 (SAS institute, Cary, NC, USA).

## 5. Results

The characteristics of patients and controls are presented in Table 1.

CAD patients showed significantly higher BMI (26.2 ± 3.83 vs. 24.3 ± 2.25 kg·m^−2^, *p* = 0.025) and number of infarcted segments (3 [2–4] vs. 0 [0–0], *p* < 0.001) compared to controls. They also had lower heart rates (61.1 ± 8.8 vs. 67.3 ± 7.1 bpm, *p* = 0.025), lower left ventricular ejection fractions (55.4 ± 11 vs. 61.6 ± 6.1%, *p* = 0.012) and a higher LV mass index (66.5 [58.1–78.9] vs. 57.8 [52.11–61.25], *p* = 0.002).

### 5.1. Differences in Stiffness Measures between CAD Patients and Controls

Concerning aortic stiffness, cf PWV was significantly higher in patients (12.67 ± 2.86 m·s^−1^) compared to controls (9.58 ± 1.13 m·s^−1^, *p* < 0.001). While 2D PWV (10.97 ± 3.43 m·s^−1^ vs. 8.01 ± 2.05 m·s^−1^, *p* < 0.001) was also significantly higher in CAD patients than controls, aortic distensibility was lower but did not reach statistical significance. 

**Figure 3:**
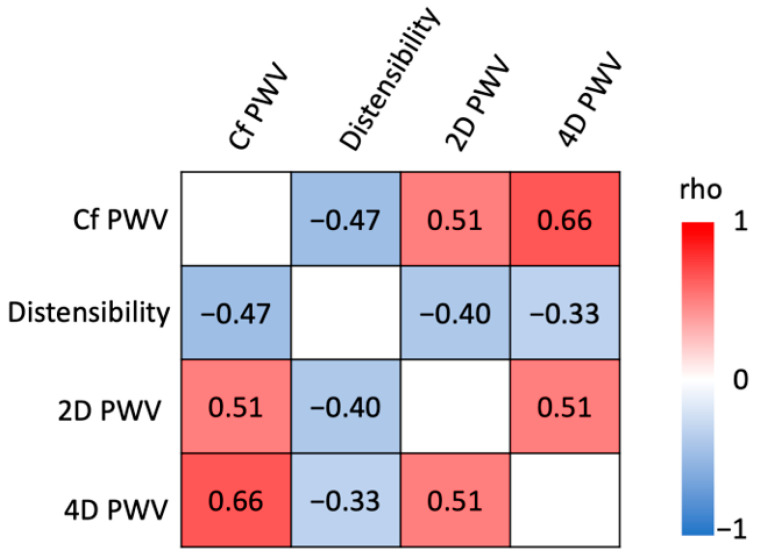
Correlation matrix between aortic stiffness estimates. Values presented are Spearman’s rho correlation coefficients.

Similarly to 2D PWV, 4D PWV was significantly higher in CAD patients compared to controls (17.3 ± 4.04 m·s^−1^ vs. 8.69 ± 2.54 m·s^−1^, *p* < 0.001). The ability of 4D PWV to discriminate CAD patients from controls was evaluated with an ROC analysis and further compared with cf PWV, aortic distensibility and 2D PWV (Table 2 and Figure 3). Overall, 4D PWV showed the best AUC (0.97), sensitivity (88.6%) and specificity (94.4%), for a threshold value of 12.86 m·s^−1^.

### 5.2. Correlations between Stiffness Indices

**Figure 4:**
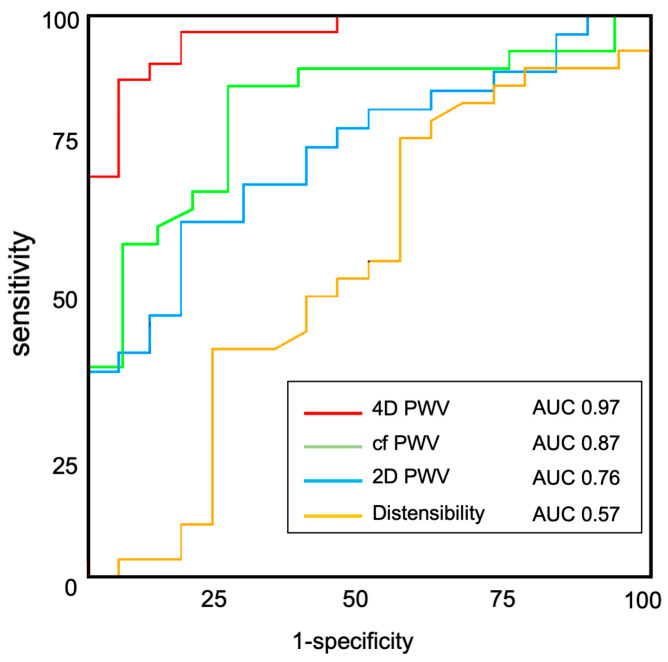
ROC curves of 4D PWV, cf PWV, 2D PWV to discriminate between coronary patients and matched controls.

From data of both groups, a correlation matrix of the relationship between all aortic stiffness estimates is provided in Figure 4, revealing a good to moderate correlation between 4D PWV and cf PWV (ρ = 0.66; *p* < 0.0001) and between 4D PWV and 2D PWV (ρ = 0.51; *p* = 0.0002). Four-dimensional PWV was less correlated with aortic distensibility (ρ = −0.33; *p* = 0.0187).

### 5.3. Relationship between 4D PWV and Clinical Parameters

In addition, a significant correlation was found between 4D PWV and the number of infarcted segments (ρ = 0.57; *p* < 0.001) and LV Mass indexed to BSA (ρ = 0.28; *p* = 0.048) (Table 3). However, such correlations were no longer significant when analyzing the groups separately. Interestingly, the correlation between 4D PWV and age was significant in the control group (r = 0.52, *p* = 0.028) but was no longer significant when considering CAD patients only or the entire population. 

## 6. Discussion

In this work, we confirmed that values of PWV in CAD patients were higher than in controls, regardless of the measurement technique. Additionally, the thoracic aorta PWV assessed from 4D flow MRI showed the best overall ability in terms of sensitivity and specificity to differentiate patients with CAD from controls as compared to 2D phase contrast PWV, aortic distensibility and cf PWV. In addition, thoracic aorta 4D PWV was well related to hypertrophic remodeling.

### 6.1. Methodological Reasons for 4D PWV Superiority in the Setting of CAD

The poor performance of aortic distensibility to differentiate patients with a severe atheromatous disease (stable CAD) from healthy controls was not a surprising result. If aortic distensibility is known to be a useful biomarker for event prediction in the early stage of cardiovascular disease [15], its performance may be highly affected at a later stage when the population is older with a stiffer aorta, since the smaller changes in the aortic lumen area over the cardiac cycle may reach the limitations of MRI spatial resolution. 

The higher performance of 4D PWV over 2D PWV might be explained by the longer aortic segment considered in the 4D flow data, by the iterative transit-time estimation along such segment that could render the overall estimate more robust to the poor temporal resolution of 4D flow MRI and by the specific slice placement that may hamper 2D PWV. Indeed, even if the 2D dataset has twice the temporal resolution of the 4D flow dataset, the PWV is estimated from a transit time calculated on a two-times shorter aortic segment.

Carotid-to-femoral PWV assessed by tonometry is less concerned by such measurement issues thanks to its high temporal resolution; however, cf PWV reflects diffuse arterial disease, while 2D and 4D PWV focus on the central aorta. The evaluation of aortic stiffness in this proximal location could explain the superiority of MRI in our population of patients with severe CAD. Our results might have been different for patients with a less central location of atherosclerosis.

### 6.2. Correlations between the Aortic Stiffness Measurement Methods

When looking at the multiple correlations between stiffness measurements, the most related were cf PWV and 4D PWV. This could be explained by the fact that both measurements look at a longer arterial segment, as compared to 2D PWV and distensibility, which are more centralized at the level of the aortic arch and the ascending aorta, respectively. The relationship between the techniques might be also affected by the precision of the measurements, explaining the poor relationship with aortic distensibility, which might be a less conclusive metric in our stiff and relatively old population.

### 6.3. Which Threshold for High PWV?

PWV is highly related to age and should always be interpreted while considering individual calendar age. Nevertheless, the ESC/ESH 2018 guidelines recommend a cf PWV cutoff value of 10 m·s^−1^ as a factor of cardiovascular risk. The 10.3 m·s^−1^ threshold of cf PWV found in our study to differentiate stable CAD from healthy controls was in line with such guidelines in favor of the reliability of cf PWV measurements in our cohort. Several studies have previously reported that PWV of the aorta was lower than cf PWV in healthy cohorts [14,20]. Our results are consistent with such findings for the control group; however, we found higher 4D PWV than cf PWV in the CAD group, resulting in an overall higher threshold of 12.8 m·s^−1^ to differentiate CAD patients from controls. 

### 6.4. Relation with the Left Ventricular Impairment

The relationship with the size of myocardial infarction, defined by the number of segments with MI, is not significant in the subgroup of patients with CAD. However, patients at the early stage of vascular disease might have had a more severe myocardial infarction due to the lack of preconditioning and collateral arteries. Similarly to previous work, arterial stiffness assessed using 4D PWV was correlated with LV ventricular remodeling [20].

### 6.5. Limitations and Perspectives

As in many studies involving PWV, no comparison with invasive measures was conducted. However, 4D flow was well correlated with cf PWV in our study, providing a validation beyond comparison between only MRI measurements. Aortic segmentation issues in the CAD group were more pronounced than in controls and would require further technical improvements through newly proposed tools that might help overcome such issues [24]. Our cohort is relatively small. However, such a small sample size is partly compensated by the homogeneity of the included patients with stable CAD, since they all had a previous myocardial infarction 1 year before and multivessel disease. Our promising results would need to be confirmed in cohorts with less severe disease.

Previous works have found relationships between arterial stiffness and prognosis in patients with coronary artery disease [33,34]. Those outcome data are lacking for 4D flow, and further works are warranted to prove that 4D PWV could do better to predict CV events in patients with or without CVD.

## 7. Conclusions

4D flow PWV outperformed other tonometry and 2D MRI stiffness measures in terms of differentiating CAD patients from age and sex-matched controls. Longitudinal studies are now needed to evaluate the prognostic performances of 4D PWV in patients with or without arteriosclerotic disease.

## Figures and Tables

**Table 1:** Baseline Characteristics.

	MI *n* = 35	Controls *n* = 18	*p*
age (y)	64.3 ± 11.7	62.0 ± 10.1	0.473
Sex Male	32 (91%)	16 (89%)	1.000
Weight (kg)	78.0 ± 14.1	71.6 ± 11.4	0.099
Height (cm)	172.2 ± 8.8	171.1 ± 8.46	0.644
BMI (kg·m^−2^)	26.2 ± 3.83	24.3 ± 2.25	**0.025**
Myocardial Segments with MI (n)	3 [2–4]	0 (0%)	**<0.001**
HR (bpm)	61.1 ± 8.8	67.3 ± 7.1	**0.025**
Central SBP (mmHg)	118 ± 12.5	116 ± 9.7	0.615
Central DBP (mmHg)	81.0 ± 10.6	82.9 ± 7.2	0.503
LV EDVi (ml·m^−2^)	58.0 [48.0–77.0]	64.9 ± 14.1	0.547
LV ESVi (ml·m^−2^)	25.0 [19.0–42.0]	24.7 ± 6.6	0.699
LVEF (%)	55.4 ± 11.0	61.6 ± 6.1	**0.012**
LVMi (g·m^−2^)	66.5 [58.1–78.9]	57.8 [52.11–61.25]	**0.002**
AA Diameter (mm)	35.1 ± 4.2	34.2 ± 3.0	0.436
DA diameter (mm)	26.7 ± 3.1	27.9 ± 3.2	0.191
Cf PWV (m·s^−1^)	12.67 ± 2.86	9.58 ± 1.13	**<0.001**
Aortic distensibility (10^−3^ mmHg)	1.71 [1.19–2.17]	1.77 [1.46–2.70]	0.375
2D PWV(m·s^−1^)	10.97 ± 3.43	8.01 ± 2.05	**<0.001**
4D PWV(m·s^−1^)	17.3 ± 4.04	8.69 ± 2.54	**<0.001**

MI: myocardial infarction. HR: heart rate. SBP: systolic blood pressure. DBP: diastolic blood pressure. AA: ascending aorta. DA: descending aorta. Cf: carotid to femoral. Baseline characteristics are provided as mean ± SD or median [inter quartile range], or *n* (percentage) as appropriate. *p* values in bold are <0.05.

**Table 2:** ROC curve analysis.

	Threshold	AUC	Sensitivity	Specificity
Cf PWV	10.30 m.s^−1^	0.87	87.5%	83.4%
Aortic distensibility	1.49 × 10^−3^ mmHg	0.57	48.5%	78.8%
2D PWV	10.23 m·s^−1^	0.76	62.1%	88.9%
4D PWV	12.86 m·s^−1^	0.97	88.6%	94.4%

ROC: receiver operating characteristics. AUC: area under the curve. Cf: carotid to femoral (tonometry). PWV: pulse wave velocity.

**Table 3:** Correlations between 4D PWV and clinical parameters for each group.

	CAD		Control		All	
	**r**	***p***	**r**	***p***	**r**	***p***
Age	0.13	0.43	0.52	0.028	0.22	0.12
cMBP	0.26	0.12	0.01	0.97	0.24	0.077
	**ρ**	***p***	**ρ**	***p***	**ρ**	***p***
Infarct size	−0.01	0.76	NA		**0.57**	<0.001
LVMi	−0.03	0.86	−0.23	0.36	**0.28**	0.048

PWV: pulse wave velocity. CAD: coronary artery disease. cMBP: central mean blood pressure. LVMi: indexed left ventricular mass. Pearson’s or Spearman’s correlation coefficients (r or ρ) are given as appropriate. Coefficients in bold when *p* < 0.05.

## Data Availability

The data presented in this study are available on request from the corresponding author.
